# Literature-Based Enrichment Insights into Redox Control of Vascular Biology

**DOI:** 10.1155/2019/1769437

**Published:** 2019-05-16

**Authors:** Magbubah Essack, Adil Salhi, Julijana Stanimirovic, Faroug Tifratene, Arwa Bin Raies, Arnaud Hungler, Mahmut Uludag, Christophe Van Neste, Andreja Trpkovic, Vladan P. Bajic, Vladimir B. Bajic, Esma R. Isenovic

**Affiliations:** ^1^King Abdullah University of Science and Technology, Computational Bioscience Research Center, Thuwal, Saudi Arabia; ^2^Vinca Institute, University of Belgrade, Laboratory for Molecular Endocrinology and Radiobiology, Belgrade, Serbia

## Abstract

In cellular physiology and signaling, reactive oxygen species (ROS) play one of the most critical roles. ROS overproduction leads to cellular oxidative stress. This may lead to an irrecoverable imbalance of redox (oxidation-reduction reaction) function that deregulates redox homeostasis, which itself could lead to several diseases including neurodegenerative disease, cardiovascular disease, and cancers. In this study, we focus on the redox effects related to vascular systems in mammals. To support research in this domain, we developed an online knowledge base, DES-RedoxVasc, which enables exploration of information contained in the biomedical scientific literature. The DES-RedoxVasc system analyzed 233399 documents consisting of PubMed abstracts and PubMed Central full-text articles related to different aspects of redox biology in vascular systems. It allows researchers to explore enriched concepts from 28 curated thematic dictionaries, as well as literature-derived potential associations of pairs of such enriched concepts, where associations themselves are statistically enriched. For example, the system allows exploration of associations of pathways, diseases, mutations, genes/proteins, miRNAs, long ncRNAs, toxins, drugs, biological processes, molecular functions, etc. that allow for insights about different aspects of redox effects and control of processes related to the vascular system. Moreover, we deliver case studies about some existing or possibly novel knowledge regarding redox of vascular biology demonstrating the usefulness of DES-RedoxVasc. DES-RedoxVasc is the first compiled knowledge base using text mining for the exploration of this topic.

## 1. Introduction

In cellular physiology and signaling, reactive oxygen species (ROS) are involved in various processes including cellular growth, gene expression, activation of signal transduction pathways, and induction of transcription factors in defense against infection [1–3]. In the vascular system, ROS play an important role in regulating endothelial function and vascular tone in physiological condition [4]. However, ROS are also involved in pathophysiological processes such as inflammation, endothelial dysfunction, and vascular remodeling in cardiovascular diseases (CVD), including hypertension [5–8]. ROS are implicated in vascular pathophysiology, leading to atherosclerosis and arterial hypertension. Moreover, ROS-generating systems were found to facilitate diseases which promote vascular pathologies, such as hypercholesterolemia, diabetes mellitus, and obesity [9]. Within the cardiovascular system (CVS), ROS have the role of signaling molecules and facilitate cellular differentiation and growth, cell migration, inactivation of NO, protein phosphorylation, and extracellular matrix production and breakdown. However, many of these effects relate to pathological changes in the vasculature [1]. ROS are produced by endothelial cells (EC), vascular smooth muscle cells (VSMC), and adventitial cells and can be generated by various enzymes [10].

We are witnessing an enormous increase in the volume of published research material, which makes it infeasible for an individual researcher or a team of researchers to track all important developments even in a specific field. This is very prominent in the biomedical domain where, in addition to the great volume of published scientific reports, the information contained in these documents is itself highly complex. For example, the following query: “(human OR mouse OR rat OR mammal∗) AND (radical∗ OR peroxide∗ OR “reductive stress” OR ROS OR “reactive oxygen species” OR RNS OR “reactive nitrogen species” OR redox OR “reduction-oxidation reaction” OR oxidative OR nitrosative OR peroxide∗ OR superoxide∗ OR detoxifi∗ OR antioxid∗ OR “polyunsaturated fatty acids” OR “arachidonic acid” OR “linoleic acid” OR hydroperoxide∗ OR “hypochlorous acid” OR peroxynitrit∗ flavoprot∗ OR xanthine oxidase∗ OR “cytochromes P450” OR catalase∗ OR sulfiredoxin∗ OR peroxiredoxin∗) AND (“angina pectoris” OR anemia OR aneurysm∗ OR angio∗ OR arter∗ OR atrial OR atrioventricular OR aort∗ OR bradycardia OR blood OR brain OR circulati∗ OR clogging OR cardio∗ OR coronary OR edema OR heart OR ishemic OR hemo∗ OR hypertension OR leukemia OR leuko∗ OR macroangiopathy OR microangiopathy OR neovascularization OR occlusion OR pericardi∗ OR sepsis OR “sickle cell” OR tachycardia OR tachyarrhythmia OR thromb∗ OR vaso OR vein∗ OR ventricular OR vascular∗ OR vessel∗)” was used to retrieve all literature specifically focused on the problems related to redox effects on the cardiovascular system in mammalian organisms. Clarivate Analytics (https://clarivate.com/) has indexed in the Web of Science (All Databases), having 36063 and 169212 scientific articles published in 2017 and in the 2013-2017 period, respectively. This clearly highlights the challenges of analyzing information even in specialized domains.

The problem of how to explore such a voluminous information pool leads to looking for ways to simplify the search for useful information. This problem is not new, and it has been clear that one needs automated systems to support analysis of information contained in published literature. The last three decades have seen numerous attempts devoted to developments in this direction. This problem is addressed through text mining. Different aspects of text mining and a complementary set of techniques for the so-called natural language processing (NLP) have been applied for the exploration of biomedical information from free text [11–23].

Different methods were used for obtaining information from free text [24–33], many based on heavy utilization of ontologies and ontology structures [28]. Also, there have been systematic efforts to combine text mining with other methods to enhance the capacity to extract useful information (for example, [30–32, 34]).

Text mining found applications in different biomedical domains [31, 35–48], for example, dealing with problems of cancers [42], disease biomarkers [47], sickle cell disease [49], tomato species [50], medicinal herbs [35], sodium channels [51], drug repurposing [37], protein analysis [40, 52], prioritization of cancer genes and pathways [41], hepatitis C virus [53], cancer risk assessment [48], associations of mutations and human diseases [54], or association of transcription factors [55].

Research in the utilization of text mining in the biomedical field has resulted in a number of applications that are accessible online, such as [56–79]. These demonstrate the increasing value of applying text mining to the biomedical field.

In this study, to support research in redox biology and its effects on CVS, we developed an online knowledge base (KB), DES-RedoxVasc (http://www.cbrc.kaust.edu.sa/des-rv), which enables exploration of information contained in biomedical scientific literature focused on redox control of vascular systems in mammals. We provide examples of DES-RedoxVasc use.

## 2. Exploration System

### 2.1. Server Architecture and Underlying Systems

DES-RedoxVasc is a publicly available visual, interactive, topic-specific literature exploration system that was developed using an upgraded version of the DES system originally developed by some of the coauthors of this report (VBB and AR) and was used as the underlying framework for several published topic-specific KB (different versions) [24, 49–55, 58, 62, 65, 68, 70, 72, 78, 80–83]. The KB is implemented and hosted on a CentOS-7 operating system. Results are provided using Apache web server version 2.4.6. A local MongoDB (2.6.11) database stores the literature repository which comprises open-access PubMed and PubMed Central articles, and the KB index and related tables are stored on a PostgreSQL (9.2.15) database. Apache Lucene was used to index the documents. Various programming languages/tools were used to develop the KB including: JavaScript, JQuery 3.0.0 C/C ++ (gcc 4.8.5), Java (OpenJDK 1.8.0_91), Perl v5.16.3, and PHP 5.4.16. DES-RedoxVasc is functional across commonly used web browsers (Windows, Linux, and Mac OS platforms) and was specifically tested for Firefox, Safari, and Chrome. The DES workflow has been described earlier [54].

### 2.2. The Literature Corpus and Dictionaries Incorporated into DES-RedoxVasc

The MongoDB literature repository contains only documents that are tagged as open access, which means that they are freely amenable to text mining. Thus, to create the literature corpus to be analyzed, the local MongoDB repository, last updated on September 03, 2018, was queried for all topic-specific PubMed and PMC articles. The same query used to query Web of Science (All Databases) above was used to create the literature corpus. The literature index server is designed to match the query to the titles, abstracts, and full-text article when available through the PMC set. The query retrieved 233399 articles.

Also, 28 topic-relevant dictionaries were used in this KB, of which eight dictionaries were newly compiled (see Table 1). The remaining 20 dictionaries were previously used in other KBs developed using the DES framework and in Table 1.

All dictionary concepts (see Table 2 for definitions) are normalized where possible. Normalization of concepts ensures that when concepts can be referred to by different symbols, names, or synonyms, it is always associated to a single entity (using an internal identifier) and it also ensures that concepts can be recognized through universal IDs such as NCBI Taxonomy ID, Entrez Gene ID, and UniProt ID that are regarded as trusted sources. For example, dealing with genes and proteins is frequently problematic in text mining. This is as a consequence of gene/protein names/symbols and their aliases, frequently denoting more than one gene/protein. We combined Entrez Gene (for genes) with UniProt (for proteins) nomenclatures which provide the official names/symbols/aliases routinely used. Then the normalization is applied in the DES system. The normalization of dictionary concepts improves the accuracy of concepts' enrichment estimates.

Some concepts are relevant to more than one dictionary, for example, enzymes are gene products, and it is expected that nomenclatures of these entity types would have a substantial intersection. The same goes for drugs and chemicals, drugs and antibiotics, gene functions and pathways, etc. It is worth noting that normalization is done at the dictionary level and not across dictionaries because (1) it is the semantically valid approach, as biological entities might be pertinent to, say, both chemicals and drugs, and should be viewed as such depending on the scope of the literature and the user's interest and (2) these dictionaries are used in a modular fashion independently from each other; it is not redundant to keep a reference to the same entity in two or more dictionaries. For example, a user might be interested only in drugs, and not in the more general collection of chemicals, and as such chooses only drugs for the KB annotation; therefore, they should have access to all drugs that are also part of the chemical dictionary. This also applies when doing dictionary specific searches within the same KB. It is not however acceptable to have redundant concepts within the same dictionary.

The literature corpus and 28 dictionaries were used for concept document mapping. The concept document mapping results were then used to statistically determine enriched concepts and enriched pairs of concepts.

#### 2.2.1. Enriched Concepts

In a KB, concepts could be statistically enriched or not. If they are enriched in the KB, this is based on their abundance in the KB corpus which should be greater than one would expect as compared to the rest of the PubMed/PMC literature. The frequency of the concept across the entire literature is indicative of the expectation of its frequency in any randomly selected sample from the literature. A concept is enriched when its frequency in the KB is significantly higher than the expected frequency. To quantify determination of which concepts are enriched, a concept has to have a *P* value < 0.05 in the DES-RedoxVasc corpus when compared to the complete set of PubMed Central and PubMed articles in our local repository; in this manner, concepts most relevant to the KB are identified. The *P* value was calculated based on the Benjamini-Hochberg procedure to correct for multiplicity testing. Note that this *P* value is also known as a false discovery rate (FDR).

#### 2.2.2. Enriched Concept Pairs

Pairs of enriched concepts are considered enriched for association by considering the abundance of their cooccurrence as compared to the individual occurrence of concepts that form the pair. So, for example, if two concepts occur 100 times each and they cooccur 90 times, there is a high chance that they are associated, because they each occurred with the other concept 90% of the time. The situation is of course not typically symmetric, but the example is just for clarification. The resulting enriched pairs of concepts may or may not be directly associated; however, the more a pair is enriched this way, the higher the probability for the association between the two concepts.

Using cooccurrence as a proxy for semantic relatedness, or association, is a well-established, if not the dominant, approach to semantic analysis and association extraction and is by no means particular to DES. PMI (pointwise mutual information) and cosine distance from Word2Vec embeddings are some of the mainstream examples of such an approach. Establishing association between two biomedical entities from the text in a biologically meaningful way (e.g., causality, inhibition, and coexpression) is however a much more challenging task, that is, the subject of much research pertinent to the more general question of NLU (natural language understanding). Focusing on one type of association, with certain simplifying assumptions, can render the task of targeted association extraction more amenable to computation, but this is not the purpose of our explorative system.

The total number of statistically enriched concepts from all 28 dictionaries used is 101938. The number of enriched concepts per dictionary is provided in Table 1. The total number of statistically enriched pairs of concepts that are themselves found statistically enriched is 5631393. The literature corpus, 28 dictionaries, enriched concepts, and enriched pairs of concepts were integrated to create DES-RedoxVasc. The resulting network of concept pairs was also embedded in a high-dimensional semantic space, therefore enabling the computation of semantic similarity between any two concepts within the KB.

#### 2.2.3. Semantic Similarity

This similarity is a metric which establishes the likeness or closeness of two concepts in terms of their potential meaning. Semantic similarity can be the result of semantic relatedness, such as synonymy, antonymy, and hypernymy. For example, tall and short are semantically similar even though they are antonyms because they both share the semantic dimension of “height.” Semantic similarity within DES is calculated as the cosine distance between two concept embeddings (vector representations in a latent semantic space). These embeddings are obtained using a skip-gram Word2Vec model trained on the DES-RedoxVasc literature corpus with normalized concept annotation. Therefore, the underlying assumption for semantic similarity in DES is concept cooccurrence, but not necessarily direct cooccurrence.

## 3. DES-RedoxVasc Overview and Case Studies

DES-RedoxVasc allows oxidative control and vascular system-related literature to be easily explored using terms and associations that are determined to be statistically enriched in topic-specific publication. Briefly, these enriched terms/concepts can be explored using the “Enriched Concepts” (Enriched Terms) link or via the “Enriched Pairs” (Enriched Term Pairs) link that provides enriched cooccurring concepts. Concepts are regarded as cooccurring based on their cooccurrence in the text within a 200-character distance from each other. However, DES-RedoxVasc only reports the portion of cooccurring concepts (pairs of concepts) where pairs are statistically enriched, thereby increasing the probability that the reported associations could have “biological relevance.” However, “biological relevance” is left to the user to check on by exploring the actual related literature provided through the interface. So, if genes or proteins keep cooccurring with a particular disease or process much more frequently than is statistically expected, then we assume that these genes or proteins are deemed to be important to the disease pathology or process (also refer to Enriched Concept Pairs).

Users can also use the “Column visibility” tab in these links to explore enriched terms using ranking options for the false discovery rate (FDR), density, kb_frequency, and bkg_freq. Also, concepts are color coded to indicate the dictionary from which the concepts are retrieved.

Moreover, each concept is linked to a clickable box through which the “Network” and “Term Co-occurrences” links can be examined. Detailed description is provided in [72]. There is also the “Literature” link that allows users to explore the literature in DES-RedoxVasc (PubMed abstracts and PMC full-text articles) and the “Network” link that allows users to explore and generate networks of enriched concept pairs. This version of DES also provides a new link named “Semantic Similarity.” Users are also provided with a “Software Manual” on the “Home” page of DES-RedoxVasc. Below, we provide several examples wherein a range of biomedical entities are used to develop insights into redox control in vascular systems.

### 3.1. Example 1: Finding the Relevant Concepts of Different Categories Using “Enriched Concepts” View

One rather simple but useful use of DES-RedoxVasc is a possibility to quickly find some of the most relevant concepts related to redox processes in CVS. For this, one can choose the “Enriched concepts” view button (on the left side). Then the page will show the list of most characteristics concepts from all dictionaries as found by the system. If one wants to see the most enriched concepts from a specific dictionary, this is possible by selecting the dictionary from the dropdown menu from the right side. As the inspection of these most characteristic concepts will show, most of them are very clearly related to the topic that we study. In the following, we examine such singled-out genes/proteins and microRNAs in more details.

Oxidants classified either as ROS [109, 110] or reactive nitrogen species (RNS) [109, 110] are generated through the cells' normal metabolic processes as well as exogenous factors such as atmospheric pollutants and irradiation. These oxidants play important physiological roles in cell maintenance and are considered not to harm the human body when oxidant-antioxidant levels are relatively in equilibrium [111]. However, in cases where the levels of these oxidants exceed the levels of antioxidants, oxidative stress (OS) is triggered [112]. To counteract this state of oxidative stress, the cells increase antioxidant production to reestablish redox homeostasis [113, 114]. However, in contrast to the oxidative mechanisms, excess levels of antioxidants lead to excess reducing equivalents of glutathione (GSH), NADPH, and NADH that depletes ROS and triggers reductive stress (RS) [115]. This state of chronic reductive stress stimulates an increase in the production of oxidants only to establish an oxidative stress state that is eventually driven back to the reductive stress state. Thus, excess antioxidant agents may also induce prooxidant effects [116].

These counter mechanisms describe the general processes that govern redox control. Moreover, the lack of redox control in the form of prolonged oxidative or reductive stresses has been linked to several disease states [117–119] including cardiovascular diseases.

**Figure 1:**
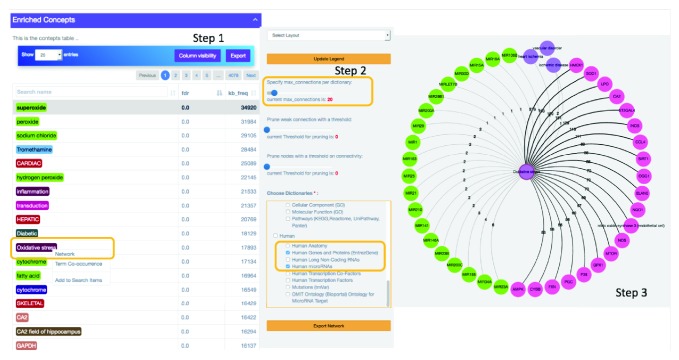
Using DES-RedoxVasc to find out potential connections between the concepts. The purple circles, the pink circles, and the green circles mark the “CVDO Ontology (BioPortal) Cardiovascular Disease Ontology” dictionary, the “Human Genes and Proteins (Entrez Gene)” dictionary, and the “Human microRNAs” dictionary, respectively. Based on the cooccurrence frequency, the color of edges can go from black (strong association) to grey (weaker association). The number of documents that link the potentially associated nodes is displayed on each edge.

Thus, we start exploring the efficacy of DES-RedoxVasc to retrieve established associations through the “Enriched Concepts” link (see Figure 1 and also see the “‘Published Examples” link for a more detailed description of how examples were generated).

#### 3.1.1. Gene/Protein Associations with “Oxidative Stress”

Figure 1 shows that the gene/protein nodes are connected with “Oxidative stress” by a large number of articles. To confirm that the genes/proteins nodes and microRNA have true associations retrieved by DES-RedoxVasc, we checked the literature suggested by DES-RedoxVasc. Li et al. demonstrated that eNOS knockout mice exhibit cardiac aging prematurely and early mortality [120]. In line with this finding, Zanetti et al. used aortae of rats (old and young) to demonstrate that the activated inducible nitric oxide synthase (iNOS), impaired SOD1 activity, and increased OS are associated with vascular aging. They also showed that caloric restriction blunts oxidative stress, reduced iNOS expression, and increased SOD1 activity [121]. They further reported that SIRT1 expression remains unchanged. However, it has been shown that human coronary arterial endothelial cells treated with resveratrol induced SIRT1, as well as upregulated eNOS in a SIRT1-dependent manner [122]. Also, OS induced with SOD1 deficiency triggers oxidatively modified CA2 to accumulate in erythrocytes [123].

ROS is also produced in normal airway epithelial cells stimulated with human neutrophil elastase (also known as HNE or ELANE) [124]. It was also shown in a large gene set that Nrf2 binds to the antioxidant response element (ARE) (including glutamate-cysteine ligase (GCL), NAD(P)H-quinone oxidoreductase 1 (NQO1), heme oxygenase-1 (HMOX1), which encodes HO-1, and thioredoxin reductase 1 (Txnrd1)) to alleviate oxidative stress [125]. Thus HO-1 was shown to play a key role in oxidative stress-related pathologies such as CVDs and atherosclerosis [126]. OGG1 repairs DNA damage induced by OS, and an OGG1 (rs1052133) polymorphism has been associated with atherosclerosis [127] and CVD [128] risk.

All genes/proteins from Figure 1 had an association with “oxidative stress” except LPO. The reason is that LPO in the text was used to refer to lipid peroxide instead of “lactoperoxidase.” Despite ELANE (with one of its synonyms being HNE) and CA2 being associated with “Oxidative stress,” in most of the articles that putatively linked these concepts to “Oxidative stress,” HNE refers to the peroxidation by-product 4-hydroxy-2-nonenal instead of the human neutrophil elastase gene or product and CA2 refers to calcium. These examples illustrate a limitation of text mining caused by multiple meanings of the same symbol.

#### 3.1.2. MicroRNA Associations with “Oxidative Stress”

On the other hand, if we look at nodes that are connected by a small number of articles such as the nodes for microRNAs in Figure 1, Step 3, we find “MIR23A” [129], “MIR34A” [130], “MIR155” [131], “MIR210” [132], and “MIR106B” [133] being associated in our KB with “oxidative stress” via “6,” “4,” “3,” “2,” and “1” articles, respectively.

**Figure 2:**
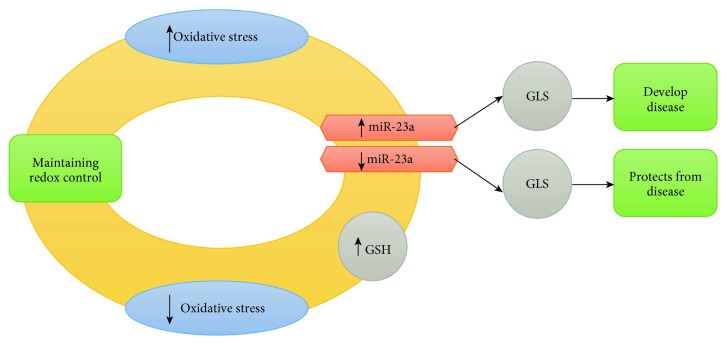
An overview of microRNA in redox control linked to maintaining a healthy state and contribution to redox dysfunction impacting different diseases.

The literature focused on “MIR23A-” (miR-23a-) revealed areas of research that may increase our insight of miR-23a-related redox control in various diseases. Dubois-Deruy et al. demonstrated that SOD2 is increased in the left ventricle after heart failure in rats, as well as miRNAs (miR-222-3p, miR-23a-3p, and miR-21-5p) targeting SOD2 [129]. They further demonstrated that left ventricular remodeling postmyocardial infarction in REVE-2 patients [134] exhibits high levels of these SOD2-targeting miRNAs. In line with this finding, it was demonstrated that inhibiting oxidative stress-induced miR-23a (MIR23A) reduces degeneration of retinal pigment epithelium (RPE) cells [135]. They further demonstrated that glutaminase (GLS) is a direct target of miR-23a and oxidative stress in miR-23a-overexpressed RPE cells is alleviated by GLS expression. This is interesting as GLS converts glutamine to glutamate, the precursor needed for synthesis of the antioxidant glutathione (see Figure 2).

Figure 2 depicts an overview of how redox control contributes to maintaining a healthy state and how redox dysfunction contributes to different disease states. When an increase in OS is coupled with the inhibition of the oxidative stress-induced microRNA, antioxidant synthesis is increased which reduces the oxidative stress back to the redox “homeostasis” state. Conversely, redox dysregulation in the form of increased expression of microRNAs inhibits antioxidant synthesis possibly leading to a disease state. This contradicts Lin et al. who, instead of inhibition, suggested the expression of miR-23a is required for the maintenance of healthy RPE cells [136].

### 3.2. Example 2: Hypotheses and Potentially New Insights Derived through the Use of DES-RedoxVasc

#### 3.2.1. Hypothesis 1: Heart Failure May Occur in Response to Oxidative Stress

**Figure 3:**
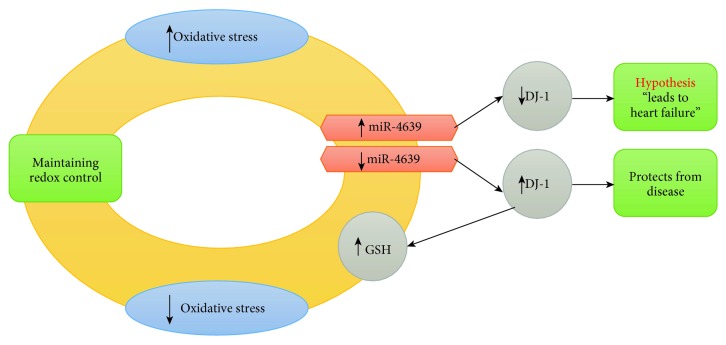
An overview of how oxidative damage may lead to heart failure.

On the page “Enriched pairs,” “Oxidative stress response” in column 1 is linked to a number of miRNAs (see column 2 when the “Human miRNAs” dictionary is selected), among which there is “MIR4639” (hsa-miR-4639). We checked the FARNA database [137] for hsa-miR-4639 and found that this miRNA is expressed in the heart [137]. Furthermore, FARNA suggests that hsa-miR-4639 is implicated in heart failure. On the other hand, Chen et al. demonstrated that increased levels of hsa-miR-4639 in plasma leads to downregulation of the DJ-1 protein activity in patients with Parkinson's disease [138]. Moreover, they demonstrated that miR-4639-5p directly binds the DJ-1 transcript at its 3′UTR that results in the downregulation of the DJ-1 protein activity. This is interesting, as oxidative stress activates DJ-1 and DJ-1 is shown to inhibit alpha-synuclein aggregate formation that leads to Parkinson's disease [139]. The relationship between miR-4639 and oxidative stress is via DJ-1, as the Nrf2-regulated antioxidant defense mechanism is impaired when levels of DJ-1 are decreased [140]. DJ-1 has also been shown to protect the heart against oxidative damage. That is, Billia et al. demonstrated that DJ-1 (with synonym PARK7) protects murine hearts against oxidative damage [141]. DJ-1 was also shown to protect the heart from ischemia-reperfusion injury [142, 143]. Moreover, the work of Li et al. shows that miR-4639 is almost 3-fold overexpressed in chronic heart failure patients compared to the control group [144]. All this leads us to the following hypothesis (see Figure 3): “overexpression of miR-4639 in the heart downregulates DJ-1 that protects the heart from oxidative damage, which may be one of the causes leading to heart failure.”

#### 3.2.2. Hypothesis 2: Vascularization Redox Is Relevant to Alzheimer's Disease

**Figure 4:**
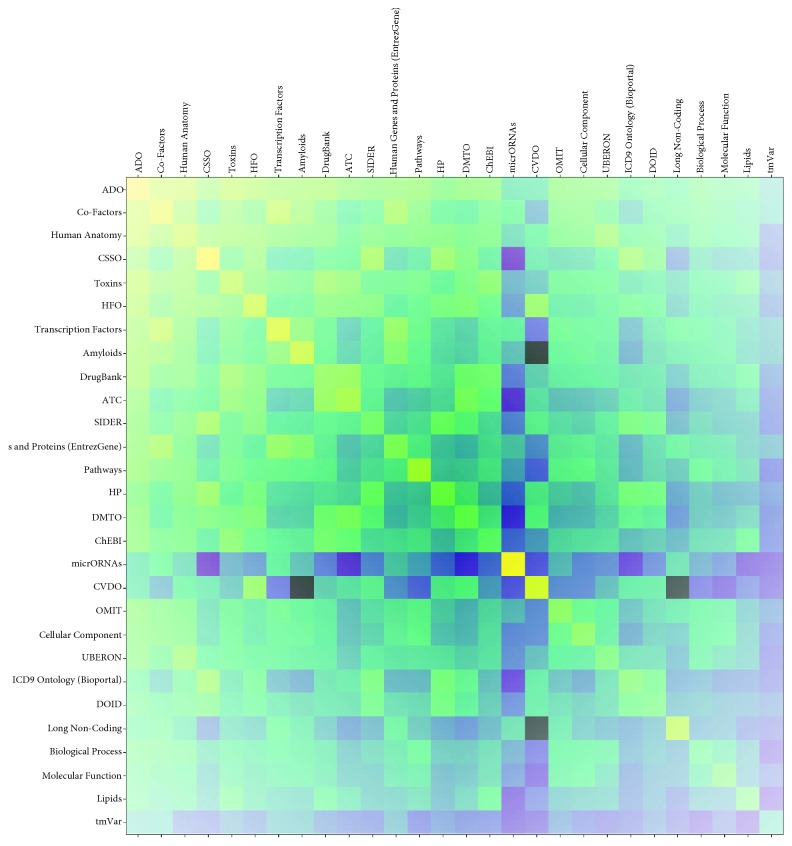
An illustration of dictionary connectivity based on concepts mapped to the analyzed corpus. Log normalized values of the number of pairs divided by the multiplication of the total amount of enriched terms in both dictionaries are displayed. The highest displayed value is -3.4 shown in bright-yellow, and the lowest value is -11.5 shown in dark purple. White is equal to the value of 0 when no pairs of concepts were found enriched for such combination of dictionaries. Rows and columns are sorted according to the total sum of enriched pairs of the dictionary, with ADO ontology having the highest normalized number of pairs.

In search of novel insights, it is also useful to look at the concepts from different dictionaries that are associated with each other. For this analysis, we looked at all connections/association found between concepts in DES-RedoxVasc.  Figure 4 shows the interconnectedness of the dictionaries with themselves and with the other dictionaries based on the cooccurring concept pairs, in the form of a heatmap. As shown in Figure 4, after normalization, the concepts from the ADO dictionary have the most connections to concepts from other dictionaries. This might seem surprising, but within the field of Alzheimer's disease research, vascularization is intensely researched as a mechanism for the disease development, with some researchers proposing that it is primarily a vascular disorder rather than a neurodegenerative disease [145]. However, since this link is based on the analysis of literature focused on redox effects to CVS, this implicitly suggests that redox-related vascular disorders may link to Alzheimer's disease. We take this observation based on Figure 4 cautiously, as the number of concepts included in different dictionaries varies as well as the coverage of a particular domain by these concepts. So, it also could be that the quality of the ontologies from which we derived some of our dictionaries is affecting the heatmap in Figure 1. In any case, it was interesting to observe potential support for the hypothesis on a link of vascularization to Alzheimer's disease.

#### 3.2.3. Hypothesis 3: ZFAS1 May Play a Role in the Fine-Tuning of the Oxidative Stress-Responsive miR-27B

**Figure 5:**
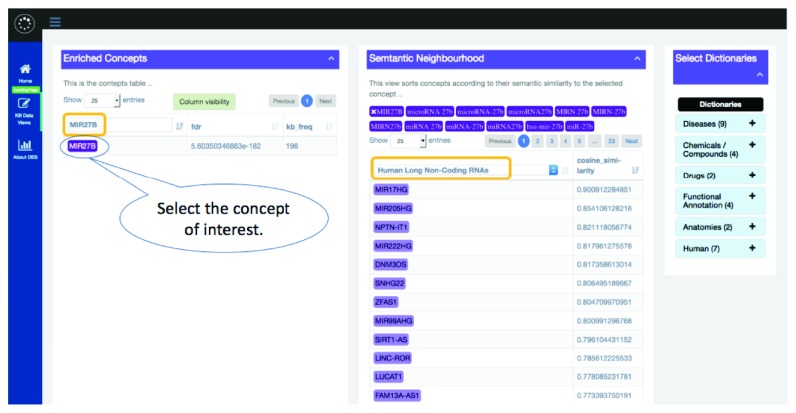
Using DES-RedoxVasc to find out potential connections between the concepts using semantic similarity.

In search of novel insights, we also looked at the associations of concepts based on semantic similarity using the “Semantic Similarity” link (see Figure 5 and also see the “Published Examples” link for a more detailed description of how examples were generated). One of the semantic similarities (similarity > 0.8) established by DES-RedoxVasc is between miR-27b and long non-coding RNA, ZFAS1. Xu et al. demonstrated that collagenase-induced intracerebral hemorrhage (ICH) in the rat brain reduces the expression of the oxidative stress-responsive miR-27b. It was also shown that overexpression of miR-27b reduced expression of Nrf2, SOD1, Hmox1, and Nqo1 and that miR-27b targets Nrf2 mRNA directly. They further demonstrated that miR-27b inhibition promotes the opposite effects, such as activation of the Nrf2/ARE pathway and reduced OS; these effects are blocked by Nrf2 knockdown [146]. Thus, miR-27b is reduced to reestablish redox homeostasis. The dysfunction of this mechanism leads to vascular diseases. That is, it was demonstrated that when miR-27b overexpresses, it induces cardiac dysfunction and hypertrophy in mice [147]. Also, Signorelli et al. demonstrated that the levels of miR-27b, miR-130a, and miR-210 are increased in patients with peripheral artery disease when compared to healthy controls [148]. However, miR-27b has not been linked to ZFAS1. Despite that, this link may be correct as ZFAS1 is predicted to bind hsa-miR-27b-3p using the DIANA tool, LncBase Predicted v.2 [149].

Current research to a certain extent supports this hypothesis, as Pan et al. reported overexpression of ZFAS1 in gastric cancer (GC) serum and tissue samples and demonstrated that ZFAS1 knockdown inhibits the proliferation and migration of GC cells by suppressing cell cycle progression and apoptosis [150], while Chen et al. demonstrated that miR-27b is downregulated in GC and show miR-27b to be a potential GC biomarker. Moreover, they show that miR-27b functions as a tumor suppressor in GC by targeting VEGFC [151]. This shows a possible inverse relationship between ZFAS1 and miR-27b. Moreover, Shin et al. report the risk of ischemic stroke and coronary heart disease incidence in GC patients [152]. ZFAS1 was also determined to be a potential biomarker for coronary artery disease/acute myocardial infarction [153]. Lyu et al. also showed ZFAS1 to be upregulated in rats with traumatic brain injury [154]. This shows that miR-27b has been linked to OS and vascular disease and that ZFAS1 has been linked to vascular disease but its possible role in the fine-tuning of miR-27b in these pathologies have not been explored.

## 4. Discussion and Concluding Remarks

DES-RedoxVasc allows for exploration of numerous associations between different concepts as they are found in the analyzed literature. Over 5.6 million such associations have been identified by DES-RedoxVasc. These potential concept associations are based on the cooccurrence of the concepts in the text placed relatively close to each other (up to a 200-character distance). Moreover, these associations are found statistically enriched in the analyzed literature with FDR < 0.05 and are made of concepts that themselves are statistically enriched in the same document set with FDR < 0.05, compared to documents in the background. Users can evaluate if such association found is meaningful by inspecting the text from where the association is derived. Another set of associations is between any of the individually enriched concepts and statistically enriched concepts that are semantically similar to them. In total, there are over 10 billion such associations found in the analyzed documents. Usually, when similarity between concepts is high, i.e., >0.75, such associations appear mostly meaningful, which reduces the number of concept pairs to an estimated 50 million.

Being primarily based on the text mining approach, DES-RedoxVasc carries all shortcomings of text mining. As we used dictionaries of terms related to different categories of concepts, the quality and completeness of these dictionaries affect the results. If a term that represent a synonym of a concept or the concept itself is not present in the dictionary, the system will not be able to identify it in the text. Also, some terms are “promiscuous” as they are very common and thus do not convey significant information. That is, promiscuous terms are terms which have very high connectivity in the knowledge graph. This is in turn due to their high frequency, because the more frequent a term is, the greater the probability for it to cooccur with more concepts. Usually, promiscuous terms have a broad semantic coverage like “function” or “disease.” Term ambiguity can also result in term promiscuity, such as the use of the term HAND or PDF as a gene symbol. Promiscuous terms might have thousands of edges, where every single edge might refer to thousands of cooccurrence hits within the annotation. Consequently, they inflate the index and the knowledge graph and therefore pose more demands on computation. More importantly, they affect the quality of extracted information and any inferences thereof, because they affect the very topology of the knowledge graph and act as high centrality hubs, creating short paths between concepts which are not otherwise associated. For example, the term “disease” can potentially link most disease concepts which are not necessarily linked, the same for pathological mutations, pathological microorganisms, etc., which are all related to the concept of disease. Removing promiscuous terms restores the intended topology of the knowledge graph. Pair enrichment provides another corrective layer for cases where promiscuous or irrelevant concepts seeped through the dictionary cleaning phase.

Computationally, to understand the improvements gained by removing these terms, we refer to the concept of term frequency distribution and in particular to Zipf's law, which establishes that a term frequency and its rank (within a descending frequency-ordered list of terms within a corpus) obey a simple power law. The main consequence of this law is that a very small proportion of top-frequency-ranked terms (usually promiscuous in a biological context) account for a substantial amount of the text (in our case, the annotation and the knowledge graph). In our latest dictionary cleaning process, the removal of 0.1% of such high-frequency terms resulted in reducing the annotation size by a third.

An additional observation is that the Cardiovascular Disease Ontology (CVDO) on the other hand does not seem to resonate well within the knowledge base, having relatively few connections, despite being conceptually of central importance. Compared to CVDO, the Heart Failure Ontology (HFO) is much better connected to the other ontologies that we used. It is possible that this is the consequence of relatively incomplete CVDO that may need some improvements if it is to show the full usefulness in text mining tasks.

Despite these limitations, the examples provided hereby as “case studies” demonstrate that the KB can be useful and that the user-friendly interface allows users to easily navigate and explore information in the KB. The DES-RedoxVasc KB literature and dictionaries will be updated biannually, and the KB will be updated accordingly.

## Figures and Tables

**Table 1:** Dictionaries used in DES-RedoxVasc with data source references.

Dictionary	Enriched unique terms in the KB	Status
*Chemicals/compounds*		
Chemical Entities of Biological Interest (ChEBI) [84]	17981	
Toxins (T3DB) [85]	2083	
Lipids (lipids maps) [86, 87]	2852	
Amyloids (Human and Mouse); compiled in-house	393	Newly compiled
*Functional annotation*		
Biological Process (GO) [88]	5438	
Cellular Component (GO) [88]	1125	
Molecular Function (GO) [88]	1755	
Pathways (KEGG [89], Reactome [90], UniPathway [91], and PANTHER [92])	1445	
*Diseases*		
DOID Ontology (BioPortal)—Human Disease Ontology [93]	3467	
ADO Ontology (BioPortal)—Alzheimer's disease ontology [94]	937	Newly compiled
DMTO Ontology (BioPortal)—Diabetes Mellitus Treatment Ontology [95]	1941	Newly compiled
HFO Ontology (BioPortal)—Heart Failure Ontology [96]	1001	Newly compiled
CVDO Ontology (BioPortal)—Cardiovascular Disease Ontology [97]	53	Newly compiled
HP Ontology (BioPortal)—Human Phenotype Ontology [98]	3204	
UBERON Ontology (BioPortal)—Uber Anatomy Ontology [99]	6540	Newly compiled
ICD9 Ontology (BioPortal)—International Classification of Diseases, Version 9 - Clinical Modification [100]	688	
*Drugs*		
Drugs (DrugBank) [101]	3918	
ATC Ontology (BioPortal)—Anatomical Therapeutic Chemical Classification [102]	1991	Newly compiled
CSSO Ontology (BioPortal)—Clinical Signs and Symptoms Ontology	210	Newly compiled
SIDER (Drug Indications and Side Effects) [103]	3190	
*Human*		
Human Genes and Proteins (Entrez Gene) [104]	21858	
Human Transcription Factors [105]	1505	
Human Transcription Co-Factors (TcoF-DB) [105]	384	
Human microRNAs (HGNC and Entrez Gene) PMEDIDs for HGNC and Entrez Gene	1811	Updated
Human Long Non-Coding RNAs (HGNC) [106]	460	
Mutations (tmVar) [107]	12514	
Human Anatomy (in-house compiled)	2538	
OMIT Ontology (BioPortal)—Ontology for MicroRNA Target [108]	656	Newly compiled

**Table 2:** Vocabulary and interactive tools used in DES-RedoxVasc.

Vocabulary and interactive tools	Definition
Concepts	Biological words or phrases (e.g., inflammation, oxidative stress, and hydrogen peroxide) found in this topic-specific literature, organized into thematic dictionaries, and used to mine the literature
Concept Pairs	Cooccurring “Enriched Concepts” (e.g., cell fate determination and TAL1; Wnt receptor and CELSR2; and *BMP2K* and coronary artery endothelial cell) that may or may not have a biological association/connection
FDR	“To be enriched, a concept or a pair has to have an FDR (false discovery rate) < 0.05 in the DES-RedoxVasc corpus. The FDR is obtained by correcting the enrichment *P* values for multiplicity testing based on the Benjamini-Hochberg procedure”
Literature	Provides the literature set used in the development of this KB
Network	A tool for the visualization of concept associations as a graph of interlinked nodes
Concept Co-occurrences	A list of concepts which cooccur in the literature with the concept in question. Concepts are regarded as cooccurring in the text if they are within a 200-character distance from each other (refer to rationale below). Only enriched pairs are shown in this list
Knowledge base	A store of information or data that is available to draw on
Dictionaries	A set of topic-specific vocabularies made up of words or phrases used for the purpose of text mining
kb_frequency	Frequency of a concept within the KB literature corpus
bkg_freq	Refers to background frequency: frequency of a concept within the whole PubMed/PMC literature corpus
Density	KB frequency divided by background frequency

## Abbreviations

CVS: Cardiovascular system
CVD: Cardiovascular disease
CVDO: Cardiovascular Disease Ontology
EC: Endothelial cells
FDR: False discovery rate
GSH: Glutathione
GCL: Glutamate cysteine ligase
HFO: Heart Failure Ontology
KB: Knowledgebase
lncRNA: Long non-coding RNA
miRNA: MicroRNA
NLP: Natural language processing
ncRNA: Non-coding RNA
Nrf2: Nuclear erythroid 2-related factor 2
OS: Oxidative stress
PMI: Pointwise mutual information
RNS: Reactive nitrogen species
ROS: Reactive oxygen species
RS: Reductive stress
VSMC: Vascular smooth muscle cells.
